# Single RGB Image 6D Object Grasping System Using Pixel-Wise Voting Network

**DOI:** 10.3390/mi13020293

**Published:** 2022-02-13

**Authors:** Zhongjie Zhang, Chengzhe Zhou, Yasuharu Koike, Jiamao Li

**Affiliations:** 1 Institute of Innovative Research, Tokyo Institute of Technology, Yokohama 2268503, Japan; koike@pi.titech.ac.jp; 2 Bionic Vision System Laboratory, Shanghai Institute of Microsystem and Information Technology, Shanghai 200050, China; chengzhe.zhou@foxmail.com

**Keywords:** 6D pose estimation, real object judgment, pixel-wise voting network, 6D grasping robotic system

## Abstract

A robotic system that can autonomously recognize object and grasp it in a real scene with heavy occlusion would be desirable. In this paper, we integrate the techniques of object detection, pose estimation and grasping plan on Kinova Gen3 (KG3), a 7 degrees of freedom (DOF) robotic arm with a low-performance native camera sensor, to implement an autonomous real-time 6 dimensional (6D) robotic grasping system. To estimate the object 6D pose, the pixel-wise voting network (PV-net), is applied in the grasping system. However, the PV-net method can not distinguish the object from its photo through only RGB image input. To meet the demands of a real industrial environment, a rapid analytical method on a point cloud is developed to judge whether the detected object is real or not. In addition, our system shows a stable and robust performance in different installation positions with heavily cluttered scenes.

## 1. Introduction

Today, intelligent systems play a vital role in human life with the rapid development of computer science. We expect a robotic system that can autonomously search the desired object, estimate its pose, grasp it, and move it to its target position. These kinds of systems can meet human demand in aspects such as packaging logistics, industrial production, medical services, etc. With the advantage of abundant information, strong robustness and low cost, computer vision techniques are increasingly being applied in intelligent systems.

In general, a robotic grasping system contains detection, planning and controlling parts. The planning and controlling parts are well developed, while the detection parts have presented a challenge in recent years [1]. Previous research employed machine learning methods to design descriptors, and showed good performance when the object was set in a simple environment or in simulation scenes with little interference and occlusion. However, a major problem with detection occurs because strategies based on vision methods are sensitive to the actual working scene. As a result, robotic grasping systems have shortcomings such as high computation cost, high latency and low accuracy. To date, it still is a challenge to grasp a texture-less irregular object placed in a heavy-occlusion scene.

To improve the performance of the grasping system, the detection part has gained widespread attention. In the detection part, there are three main tasks: object recognition, pose estimation and grasping point location. In addition, the algorithms of pose estimation can be divided into correspondence, template, voting and regression. On one hand, correspondence methods such as scale invariant feature transform (SIFT) and spin image have a high dependence of depth sensor resolution, while template methods such as LineMod have high sensitivity to occlusion scenes. On the other hand, voting and regression methods by utilizing deep neural networks have a more robust performance.

Considering the robotic arm sensor performance and the system versatility, we input RGB images to train the pixel-wise voting network (PV-net), and utilized a depth camera to judge whether the detected object was real or not and assist grasping point detection. The main contributions are as follows:

1. By reprojecting the object model into the pose estimation space, we improve the performance of the pixel-wise voting network (PV-net) so that PV-net can operate properly under a real working scene of lighting interference and low resolution. In addition, PV-net is only in the theoretical stage so far, whereas we apply this pose estimation network in a real robotic system.
2. Based on the RGB image as the network input, it is difficult to tell the difference between the object and its photo. The native sensor of the robotic arm platform hardly meets the requirement of the RGB-D deep learning network training. To overcome this shortage, a fast and stable method is proposed to judge whether the detected object from PV-net is real or not by analyzing the point cloud captured from the depth sensor.
3. By combining the methods described above, an autonomous 6 dimensional (6D) grasping system is implemented to accomplish real-time grasping tasks in industrial packaging logistics environments where the real object is mixed with its wrapping paper photos. This stable and robust grasping system is potentially applicable to large-scale production pipelines.

**Figure 1:**
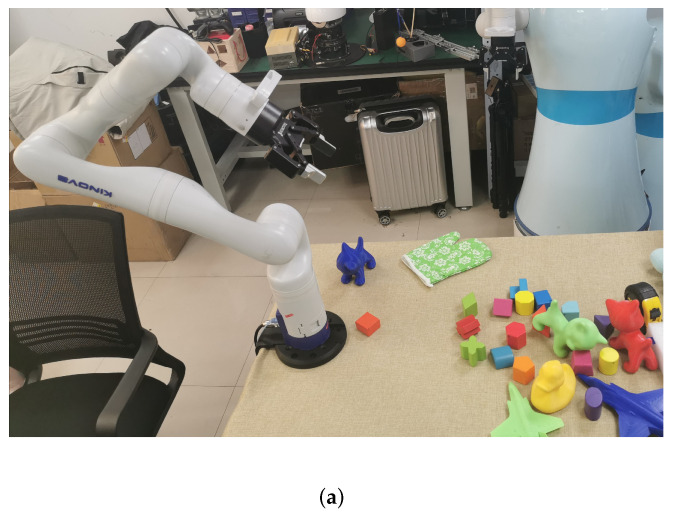

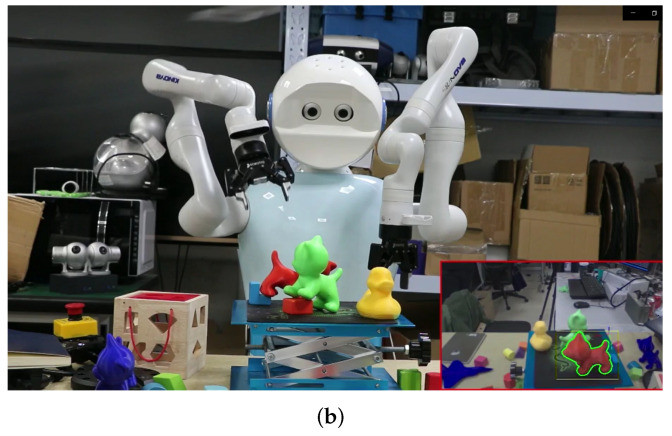
The intelligent robotic 6D grasping system working under different installation positions. (**a**) Robotic system is installed on the table; (**b**) robotic system is installed on the robot.

As Figure 1 shows, our robotic grasping system can work anywhere in the real scene, no matter the installation position. In the testing situation, when the distance from the sensor to object grows further, causing the pixel and point cloud information to be limited, the grasping system can still maintain a high capture success rate and accuracy rate of pose estimation.

## 2. Previous Research and Relative Approaches

In this part, the development of pose estimation is reviewed by listing some existing approaches. Besides, some robotic grasping systems with pose estimation algorithms are also illustrated.

### 2.1. Pose Estimation Approaches

It’s essential to apply pose estimation algorithms on robotic systems to meet the demand of different complex tasks. According to the key idea of algorithm design, the methods are described as follows:

#### 2.1.1. Correspondence Approaches

Early pose estimation methods [2,3,4,5] rely on sparse feature point matching between 2D images and 3D object models. For objects with abundant textures, local 2D key points can be detected both efficiently and accurately thus leading to robust estimation of the object pose even under cluttered scenes and severe occlusions [6]. Besides, Pavlakos et al. [7], Tekin et al. [8] and Zhou et al. [9] compute correspondence from 2D pixels to the 3D model to regress the pose value by defining and predicting the synthetic key points. However, these feature-based methods, though simple and fast, have difficulty in handling poorly textured objects and processing low-resolution images.

#### 2.1.2. Template Approaches

Template matching is perhaps one of the most favorite approaches for model-based object detection [10,11]. In general, these methods compute the distribution of image responses (measured by some distance metric) to a set of templates scanned across the entire image and look for the best match. For example, LINEMOD [12] uses synthetic renderings of a 3D object model to generate a large number of templates covering the full view hemisphere. They employ an edge-based distance metric based on stable gradient and normal features for template matching, which works well for texture-less objects, and refine their pose estimates using iterative closest point (ICP) to achieve an accurate 6D pose. Such template-based approaches can work accurately and quickly in practice. However, these methods are designed to work with RGB-D data and have high sensitivity of lighting and occlusion.

#### 2.1.3. Regression Approaches

Convolutional neural networks (CNNs) have provided efficient regression solutions in 6D pose estimation in recent years. PoseNet which applies a CNN architecture regresses a camera 6D pose with RGB images as input. Based on the building information model (BIM), various BIM-PoseNets are fine-tuned to regress the pose of real image sequences for indoor space [13,14,15,16,17,18]. Although BIM-PoseNets perform well with relatively high accuracy, they not only require 3D indoor models as prior information, but also rely on the point cloud from high quality sensors. As a result, they are not suitable for real-time robotic systems in unknown environments with heavy occlusion.

#### 2.1.4. Voting Approaches

The idea of voting methods is that the local evidence in the image restricts the possible outcome of the desired output while each image patch is used to launch a voting about the output. Therefore, the advantage of voting methods is robust in the scene of objects with occlusions. Point pair feature (PPF) is proposed to recover the object 6P pose from depth information [19]. Similarly, a generic framework named DenseFusion is proposed for a set of known objects 6D pose through the input of RGB-D images [20]. In this paper, the pixel-wise 2D key points prediction through voting based on the direction is employed to improve the robustness of our grasping system for meeting the challenges of cluttered scene and truncated objects.

### 2.2. Robotic Grasping

The rise of robotic systems has been accompanied by the development of algorithms and devices [21]. Through the application of various algorithms, robotic systems have seen great improvement in the field of automation. Three-dimensional grasping systems are implemented by multi-view point cloud [22] and single image input [11]. Through adding synthetic key points to CNN architecture, James et al. [23], Tremblay et al. [24], Wan et al. [25] and Stevsic et al. [26] also implement 6D grasping and assemble tasks. However, current research based on pose estimation does not consider orientation, or the target geometry is very simple, such as boxes or blocks, etc. Meanwhile, most grasping systems cannot resist interference from object photos. In this work, we design a real-time robust robotic system which can distinguish a real object from its photo and work properly in real cluttered scenes to grasp an irregular texture-less target.

## 3. Materials and Methods

In this section, we describe the implementation of our autonomous robotic grasping system in detail.

### 3.1. Robot Model and Perception Hardware

**Figure 2:**
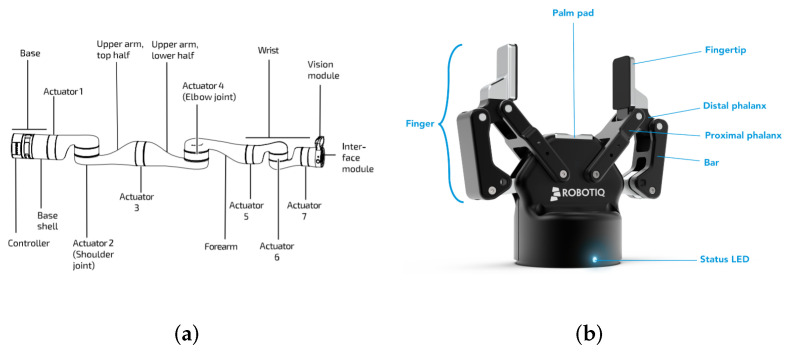
The models of the robotic arm and gripper composing the grasping system. (**a**) The kinematic model of KINOVA Gen3; (**b**) the kinematic model of Robotiq 2f.

For robotic execution, we use Kinova Gen3 (KG3), a 7-degrees of freedom (DOF) modular and adaptable robotic arm, shown in Figure 2a, to implement the task. In addition to the joints, Robotiq 2f-85, shown in Figure 2b, a parallel gripper with elastic fingertips, is attached to actuator 7 to perform grasping. The pose of the gripper’s grasping frame w.r.t. the base, which we define as the end-effector pose $T_{be}$, is known from the robot’s kinematics. The control of $T_{be}$, i.e., the absolute orientation and position of the gripper, is achieved through the inverse kinematics solver provided by Kinova.

For perception, we use the vision module of KG3, which is mounted on the top side of the interface module shown in Figure 2a. The vision module includes a color sensor (Omnivision OV5640), which streams 640 × 480 RGB images, and a stereo depth sensor (Intel RealSense Depth Module D410), which streams 480 × 270 depth maps. Both sensors were calibrated using standard camera calibration methods.

### 3.2. 6DOF Pose Estimation Based on PV-net

Given a decent point cloud of an object and a clear separation from the background, object pose estimation can be carried out using registration-based methods such as iterative closest point (ICP) algorithms and their variants. However, KG3’s native depth sensor is not capable of producing a reliable point cloud for objects that are 50 centimeters away. The low quality of its depth output significantly undermines the results of registration-based methods. To resolve this issue, we move to RGB-only methods.

PV-net [27] is a novel framework for model-based 6DoF object pose estimation. It detects objects and estimates their orientations and translations using a two-stage pipeline: CNNs are first trained to predict the 2D keypoints of the object, as 2D keypoint detection is relatively easier than direct 3D localization from the whole image, and then the object pose is estimated from these keypoints through 2D–3D correspondences with an uncertainty-driven PnP algorithm.

The robustness of PV-net stems from its vector field representation of object keypoints. During the training stage, in contrast to methods that directly regress 2D keypoint locations from image segmentation of the model object [7], which have difficulty in predicting unseen keypoints of occluded and truncated objects, PV-net trains each pixel not only to learn the semantic label that associates it with a specific object but also unit vectors that represent the direction from this pixel to every known keypoint of the object. More specifically, if $x_{k}$ is one of the 2D keypoints projected from the 3D object, the network learns a conditional vector field $v(p|x_{k})$ defined for each pixel p in a segmented region of the object where

(1) $$v\left(p|x_{k}\right)=\frac{x_{k}-p}{∥x_{k}{-p∥}_{2}}$$

represents the relative direction from a pixel to the known keypoint.

Keypoint localization using vector field representation is an overdetermined problem for PV-net, since the intersection of any two non-collinear rays that correspond to vectors $v(p|x_{k})$ and $v(q|x_{k})$ at pixel p and q that share the same semantic label would generate a hypothesis $h_{k}$ for the location of the 2D keypoint $x_{k}$. In order to recover the most confident hypothesis, PV-net implements a RANSAC-based voting scheme to compute the voting score *w* of a hypothesis $h_{k}$, where *w* is the total number of pixels p such that $v\left(p|h_{k}\right)\cdot v\left(p|x_{k}\right)$ is greater than a threshold (usually set to 0.99). Instead of naively choosing the hypothesis with the highest voting score, which can be potentially unstable, PV-net samples a large number of pixel pairs in the image and generates a discrete spatial distribution of keypoint location hypotheses $\left\{h_{k,i}\right\}$ with their voting scores $\left\{w_{k,i}\right\}$. Finally, the optimal keypoint location and its uncertainty in a statistical sense can be naturally derived from the weighted sample mean

(2) $${\hat{h}}_{k}=\frac{\sum_{i}w_{k,i}h_{k,i}}{\sum_{i}w_{k,i}}$$

and the weighted sample covariance

(3) $$\sum_{k}=\frac{\sum_{i}w_{k,i}\left(h_{k,i}-{\hat{h}}_{k}\right){\left(h_{k,i}-{\hat{h}}_{k}\right)}^{⊤}}{\sum_{i}w_{k,i}}\;.$$

During the last stage of pose prediction, PV-net repeats the above localization process several times for each predefined 3D keypoint $p_{k}$ of the object. With 2D keypoint locations $\left\{{\hat{h}}_{k}\right\}$ and associated uncertainties $\left\{\sum_{k}\right\}$ predicted for an object, PV-net recovers the 6D pose T of the object by solving a modified PnP problem that seeks to minimize the Mahalanobis instead of the Euclidean distance of the reprojection errors:

(4) $$\min_{T}\sum_{k}{\left(\Pi \left(Tp_{k}\right)-{\hat{h}}_{k}\right)}^{⊤}\sum_{k}^{-1}\left(\Pi \left(Tp_{k}\right)-{\hat{h}}_{k}\right)\;.$$

This concludes the original pipeline of PV-net.

In practice, due to subtle variations in imaging conditions and the occasionally erroneous convergence of the PnP algorithm, PV-net would sometimes produce false pose predictions that are infeasible for grasping. In order to handle these false predictions, we are inspired by the observation that the 2D object segmentation belongs to a completely separate pipeline from the part of the network on 6D pose prediction and has been shown to be very resilient to noise. Therefore, we insist that the expected projection of a 3D object based on PV-net’s 6D pose prediction must be consistent with its 2D segmentation mask, which consists of pixels that share a common label of this object.

To concretely visualize this, we consider the output from PV-net, which consists of a predicted 6D pose $T_{o}^{c}$ in the camera’s frame and a 2D segmentation mask (denoted by $M_{S}$) representing a collection of pixel coordinates that match the semantic label of a segmented object. Since the 3D model of the detected object is known, we can sample a dense point cloud (denoted by O) from its CAD model in the body-fixed frame. Then, in an ideal situation, if $T_{o}^{c}$ is perfectly correct, the projection of the 3D point cloud O to the image plane (denoted by $M_{P}$) via the camera’s projection model Π with known camera intrinsics must match with the 2D object semantic segmentation $M_{S}$. In practice, a complete overlap is impossible and a threshold σ on the percentage of overlap between two masks is set instead:

(5) $$\frac{\left|M_{S}\cap M_{P}\right|}{\left|M_{S}\right|}>\sigma ,\;M_{P}=\left\{\Pi (T_{o}^{c}p)|p\in O\right\}\;.$$

where |A| counts the total number of non-zero pixels in mask *A* and |A∩B| counts the total number of non-zero pixels in both mask *A* and *B*. During our experiments, we found that σ=0.85 is sufficient to reject most false pose predictions by PV-net.

### 3.3. Reprojection Judgment in Point Cloud

**Figure 3:**
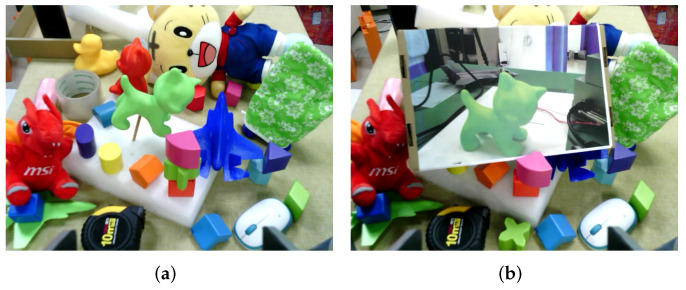
The real object and its photo in the same scene. (**a**) A real object in the scene; (**b**) a photo of the real object.

Without depth information, it is challenging to distinguish the stereoscopic target from its photo only based on RGB image input, as shown in Figure 3. We propose a fast analytical method based on point clouds to judge whether the detected object is real or not to meet the demand for a real-time system.

Based on the estimated 6D pose value, we rotate the KG3’s end effector to keep the depth sensor facing about 45 degrees downward the object, then we reproject the object’s model as a bounding box into the point cloud captured by the depth camera. Then, we can obtain the overlapped point cloud data via applying the bounding box as a pass-through filter. It is intuitive that the overlapped point cloud has a complex geometric shape when the object is real. Otherwise, the object model would appear in an unexpected position such as a very far or very close position, which means the overlapped point cloud has no data. Additionally, the shape of the overlapped point cloud would be flat in such a special condition of the object model being reprojected in the photo.

Therefore, we can directly reject the detection when there are no points in the overlapped point clouds. Then, we analyze the point distribution of the overlapped point cloud if there are points in the intersection space. The points of a photo mainly focus on the middle depth of the space, meaning that the detection result is the object’s photo. On the contrary, the points of a real object focus at the farthest and nearest ends of the intersection space. Based on this phenomenon, we slice the overlapped point cloud according to the depth and count the point number of the point cloud in each slice, use the least square method to fit a quadratic function based on the point number as (6) and (7) and determine the detection result according to shape of the curve. We reject the detection if the result of the fitted quadratic function is negative; otherwise, we accept it.

(6) $$F\left(loss\right)=\sum_{i=1}^{n}{\left[p_{i}-\left(a_{0}+a_{1}n+a_{2}n^{2}\right)\right]}^{2}$$

(7) $$P=a_{0}+a_{1}n+a_{2}n^{2}$$

where F(loss) denotes the loss function, $p_{i}$ denotes the point number of the sliced point cloud *i*, and $a_{n}$ and *n* denote the coefficient and independent variable of the fitted quadratic function *P*.

### 3.4. System Implementation

**Figure 4:**
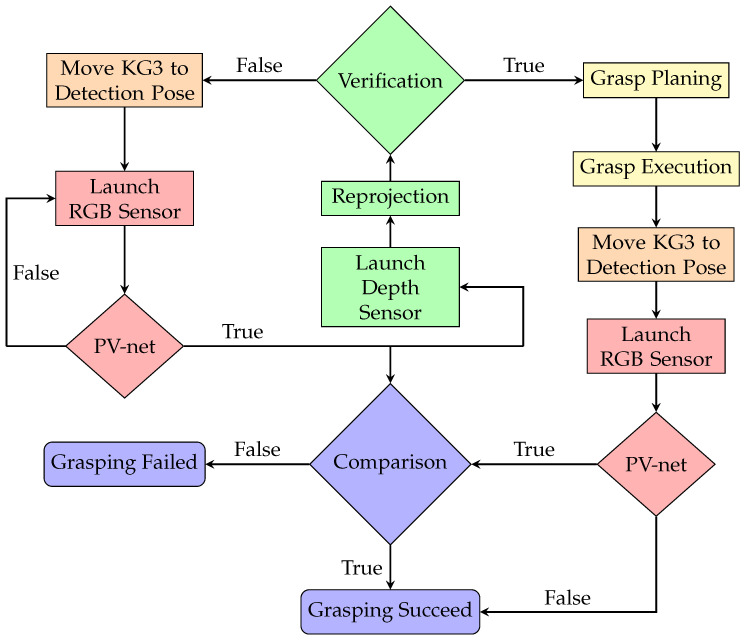
The architecture of our proposed autonomous 6D grasping system. The red parts denote the estimation of object 6D pose via PV-net, green parts denote the judgment of the object and its photos and purple parts denote the judgment of the successful operation of the grasping system.

On the basis of hand–eye calibration, PV-net reproduction, and object judgment, we design an autonomous grasping system, as shown in Figure 4. The pipeline of the grasping system to grasp a known, irregular, and texture-less object in a heavy occlusion scene is implemented as follows:

1. Move the KG3 to a preset pose which is prepared for detection.
2. Launch the RGB camera to send the 640 × 480 resolution image to the PV-net server via the Ethernet at a speed of 30 frames per second for object recognition, segmentation and pose estimation. KG3 continues to wait until the object is detected.
3. Launch the depth sensor after receiving the 6D pose value and judge whether the object is real in the scene captured by the depth sensor. If the judgment is rejected, move KG3 back to step 1.
4. Calculate and execute the grasping plan after the judgment is passed. After grasping the object and placing it to the designated location, move KG3 back to the preset object detection pose.
5. Launch the RGB camera and detect the object via PV-net again. If PV-net cannot detect the object, it means that there is no object in the scene. Otherwise, compare the previous 6D estimated pose with the current 6D estimated pose to judge whether this grasping is successful or not.

In the grasping point location, we move and rotate KG3 based on the 6D estimated pose value to keep the gripper 20 cm above the object, which is perpendicular to the object intrinsic horizontal plane. Then, we fine-tune the gripper’s position from the top view and the object’s model to locate the best grasping point. We set the minimum radius of the object as the threshold. If the Euclidean distance of two pose values is greater than the threshold, it means there is another object in the scene, or else it means the grasping task failed.

## 4. Results

In this section, we illustrate the performance of PV-net, reprojection judgment and the entire system.

### 4.1. The Performance of PV-net  Perception

**Figure 5:**
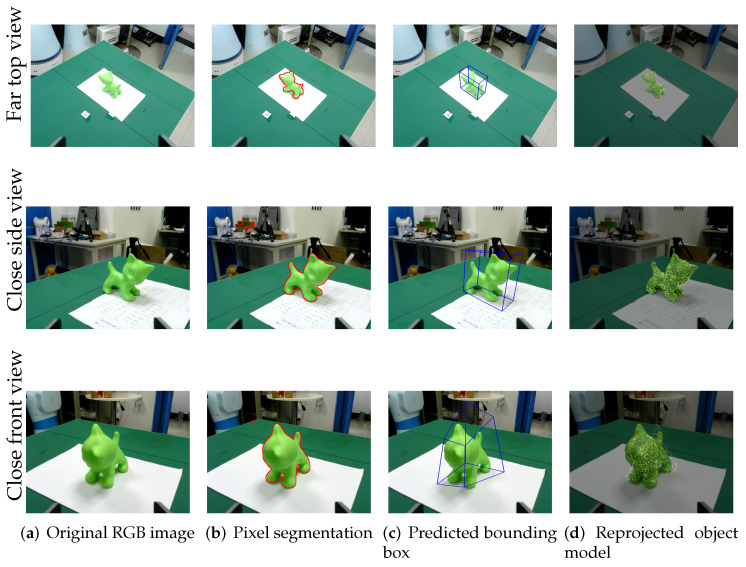
Estimating 6D pose using PV-net at different viewpoints. The target object in the original RGB image (**a**) is first segmented into “cat” labels, i.e., pixels enclosed by the red contours in (**b**), which are then processed by PV-net to generate a prediction of the object’s pose (**c**). The confidence of the predicted pose is checked against the reprojection of the object’s CAD model (**d**).

To illustrate the effectiveness of our one-shot grasping system, we took several snapshots during the pre-grasping stage, as shown in Figure 5. At each single viewpoint, PV-net as a perception front-end performs two tasks, target object detection by assigning pixel-wise labels to the entire image, e.g., the region enclosed by the red contours in Figure 5b, and 6D pose prediction of the segmented object based on pixel-wise voting, e.g., the transformed blue frames shown in Figure 5c. To reject inconsistent predictions, we sample a dense point cloud over the surface mesh of the object’s CAD model, which is then reprojected to the image plane according to the predicted pose and camera intrinsic parameters.

In our experiments, PV-net performs robustly most of the time. We notice that the segmentation network occasionally assigns the wrong pixel labels, as shown in Figure 5b, which are usually associated with specular surfaces and distant objects of green color. Thanks to the RANSAC-based voting scheme at the core of PV-net, the resulting pose prediction is not affected by these outlier pixels. Figure 5d (in particular, its top and middle rows) shows remarkable agreement between the reprojected point cloud sampled from the CAD model and the observed image projection of the 3D-printed real object, which validates the accuracy of 6D pose prediction by our trained PV-net.

We also report a few cases where PV-net yields unstable estimations, at least with our trained PV-net model. As shown in the bottom row of Figure 5, the target object is observed at a close viewpoint with the diagonal axis of its bounding box orienting towards the camera. This particular pose introduces a prominent perspective distortion to the image projection of the target object (especially the head part), which gives rise to high uncertainties in the voting histogram of the object’s 2D key points, and consequently a large variation in the convergence of PnP pose computation. The vanilla PV-net does not provide any means of detecting unstable pose estimation. Our validation step, described in Section 3.2, is designed to handle spurious pose predictions by measuring the degree of misalignment between object segmentation (region enclosed by red contour in Figure 5b) and reprojected point cloud (yellow dots in Figure 5d). If the extent of misalignment exceeds a certain threshold, then the current pose prediction of PV-net is flagged as rejected. In such a case, we would force the robot manipulator to maneuver until a better viewpoint with a more confident pose estimation is reached.

### 4.2. The Performance of Reprojection Judgment in Point Cloud

**Figure 6:**
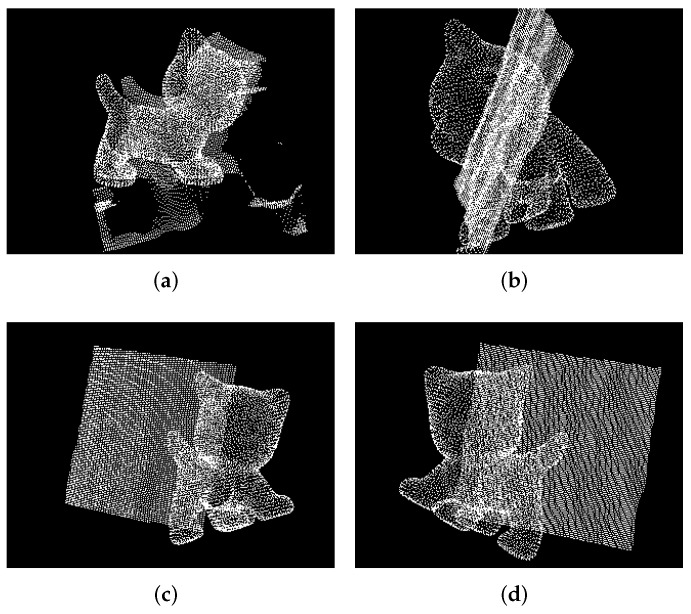
Object model reprojected into the point cloud captured by the depth sensor. (**a**) A real object in the scene; (**b**) the object model is reprojected on the photo; (**c**) the object model is reprojected behind the photo; (**d**) the object model is reprojected in front of the photo.

Here, we illustrate the function of the point cloud reprojection judgment and its performance in different parameters. There are four kinds of situations shown in the Figure 6. When the actual object exists in the point cloud scene captured by the depth sensor, it appears as a point cloud contour-like shadow around the model, as shown in Figure 6a. When the object is a photo, the reprojection model appears on the photo plane, behind the photo plane and in front of the photo plane, as shown in Figure 6b,c,d, respectively.

Employing the depth sensor as the base point, we obverse the reprojection judgment performance by randomly placing the object and its photo on the spherical surface at the same distance from the base point. The system loses its general function when the grasping distance is less than 0.3 meters, while the resolution of the depth sensor becomes low when the grasping distance is greater than 0.7 meters. On the other hand, the slice number of the point cloud also affects the performance of the reprojection judgment.

**Figure 7:**
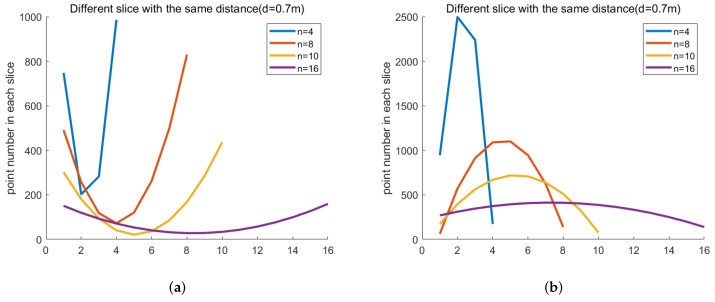
The curve of the fitted quadratic function when changing the number of point cloud slices at the furthest effective working distance (d is equal to 0.7 m). (**a**) Fitted quadratic function of real object; (**b**) fitted quadratic function of object photo.

The fitted quadratic function is shown in Figure 7 to present the difference when changing the slice number *n* at the furthest effective working distance *d*. When the slice number *n* of the point cloud decreases, the fitted curve becomes sharp because there are fewer data for fitting. The accuracy of *n* equal to 4 (84.77%) is less than *n* equal to 8 (86.68%) and 10 (96.26%), as shown on the left side of Table 1. When the slice number *n* of the point cloud increases, the fitted curve becomes flat because the discrimination between each slice point clouds is low. As a result, the accuracy of *n* equal to 16 (87.56%) is less than *n* equal to 10 (96.26%). On the other hand, we change the distance *d* when the slice number *n* is equal to 10. The accuracy rate at all distances is very high (greater than 90%), as shown on the right side of Table 1.

### 4.3. Entire System Performance

**Figure 8:**
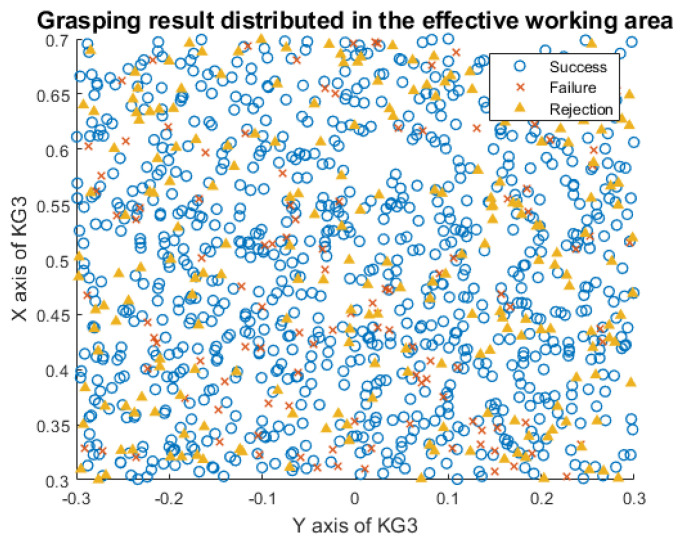
The distribution of grasping results in the KG3 effective working area. Each spot represents the real object or its photos placed in the working area. The coordinate origin denotes the installation position of the KG3. The blue circles denote the success cases of grasping a real object, while the red crosses denote the failure cases, which include the failure of object grasping and photo rejection. In addition, the yellow triangles represent the position where the KG3 rejects the object photos successfully.

Based on the architecture diagram from Figure 4, we implement the 6D grasping system. The objects and photos in a random pose are placed in a heavy-occlusion scene in a KG3 effective working area. Figure 8 shows the distribution of the grasping position and the performance of our 6D grasping system. The accuracy is calculated according to the experimental results. In the scene without target photos interference, the accuracy of the 6D grasping system reaches 95.53%. After adding target photo interference, the accuracy rate dropped slightly, to 90.42%. In addition, the failure cases of grasping mainly occur at the boundaries. KG3 tends to be stuck when the detected target appears near the effective working range, because we utilize the default scheme instead of optimizing the motion planning. This limitation of our 6D grasping system will be fixed in future work.

## 5. Conclusions

In this paper, we propose an autonomous real-time 6D grasping system which can resist interference from object photos. The deep learning method PV-net shows high performance in object recognition and pose estimation; meanwhile, the reprojection judgment shows a stable performance in real object judgment. In addition, the gripper can fine tune itself to search for the suitable grasping point via the depth camera. In conclusion, the over 95% accuracy rate proves the stability and robustness of our grasping system. In the future, we will continue to improve the intelligence of our system. For example, the function of simultaneous localization and mapping (SLAM) and obstacle avoidance will be implemented to make our system more robust in motion planning. Cooperation with other systems will also be taken into consideration.

## Figures and Tables

**Table 1:** The accuracy rate of different slice numbers (the distance ***d*** is equal to 0.7 m) and different distances (the slice number *n* is equal to 10).

Slice (*n*)	Accurate Rate (%)			Distance (***d***)	Accuracy Rate (%)		
	Object	Photo	Average		Object	Photo	Average
4	82.91	86.63	84.77	0.4 m	92.48	93.80	93.14
8	88.09	85.27	86.68	0.5 m	93.14	95.42	94.28
10	93.54	98.95	96.26	0.6 m	91.97	93.95	92.96
16	85.96	89.15	87.56	0.7 m	93.54	98.95	96.26
